# A Darwinian Perspective on Tumor Evolution

**DOI:** 10.7150/ijbs.130014

**Published:** 2026-03-30

**Authors:** Amancio Carnero

**Affiliations:** 1 Instituto de Biomedicina de Sevilla (IBIS)/HUVR/CSIC/Universidad de Sevilla, Avda. Manuel Siurot S/N, 41013, Seville, Spain.; 2 CIBER de Cancer (CIBERONC), Instituto de Salud Carlos III, Madrid, Spain.

## Abstract

Tumor evolution refers to the process by which a tumor develops and changes over time. Tumor evolution is a complex phenomenon that involves a series of stages and factors that contribute to tumor growth, progression and dissemination. These factors may include genetic mutations, changes in gene expression, interactions with the tumor microenvironment, and the immune system response. Tumor evolution can lead to the acquisition of characteristics that allow the tumor to evade the body's control mechanisms and become more aggressive. This may include the capacity for uncontrolled growth, invasion of surrounding tissues, metastasis formation, and resistance to therapy. Understanding tumor evolution is essential to develop more effective prevention, diagnosis and treatment strategies against cancer. Current research is actively studying the mechanisms involved in tumor evolution to identify new targeted therapies and treatment strategies that can address tumor heterogeneity and plasticity. In this review we provide an evolutive perspective of Cancer. From a Darwinian point of view, we present tumors as biological entities subject to similar traits to evolve and develop as natural species. This Darwinian process has strong effects in the clinical tumor as organism behavior, alone or after treatment. From understanding tumorigenesis to treatments, from cancer as disease in its interaction with the microenvironment to therapies; in this review we provide a biological perspective to better understand tumor evolution.

## Introduction

Tumor evolution is a dynamic and complex process that involves a series of genetic, epigenetic and phenotypic changes that occur in tumor cells over time. At the heart of this process lies the accumulation of genetic mutations and molecular alterations that lead to the development and progression of cancer 1, 2. The founding idea of clonal evolution was firs reasoned by Nowell in 1976 3. Nowell proposed that tumors arise from a progenitor cell and that tumor progression results from “acquired genetic variation” and clonal selection of subpopulations with growth advantages 3.

From a scientific perspective, tumor evolution can be understood as a Darwinian process within the human body. As in biological evolution, tumor cells compete with each other for resources, adapting and positively selecting genetic variants that confer survival and proliferation advantages, while the less favorable variants will tend to disappear. Genetic mutations, both spontaneous and those induced by external factors such as radiation or carcinogens, are the main drivers of tumor evolution and may grant a selective advantage for those cells containing them. These mutations can affect key genes involved in cell cycle regulation, apoptosis, senescence and DNA repair, among other fundamental cellular processes. Tumor heterogeneity (within and between tumors) caused by genomic instability is a central characteristic of tumor evolution. Within a tumor, cell subclones with diverse genetic and phenotypic profiles can be found 4. This cellular diversity may arise due to the accumulation of random mutations, as well as the selective pressure exerted by the tumor microenvironment, including factors such as hypoxia, inflammation, and interaction with stromal cells 5-7. Cellular plasticity is another crucial aspect of tumor evolution. Phenotypic changes may involve genetic alterations, although epigenetic modifications are more prevalent. Tumor cells can alter their phenotype in response to environmental cues, allowing them to adapt to changing conditions and overcome obstacles imposed by the immune system or anticancer therapy. Understanding tumor evolution is essential for the development of more effective diagnostic and treatment strategies 8.

Advances in genomic technologies and big data analysis have made it possible to identify genetic and epigenetic markers associated with tumor progression, opening new opportunities for targeted therapy and precision medicine in cancer treatment 9, 10.

While tumor heterogeneity and Darwinian clonal evolution have been widely explored, most models emphasize irreversible genetic diversification as the primary substrate of selection. In this review, we propose a complementary and integrative perspective in which tumor evolution is also driven by environmentally induced, reversible phenotypic transitions. We argue that hypoxia and other microenvironmental pressures actively promote dedifferentiation and acquisition of stem-like states, thereby dynamically reshaping the pool of selectable tumor cells. This framework positions cancer stemness and cellular plasticity not as static properties, but as adaptive traits under Darwinian selection, linking microenvironmental stress to tumor evolution, progression, and therapy resistance.

Recent conceptual frameworks have expanded classical Darwinian models of cancer evolution by emphasizing tumors as dynamic ecosystems in which genetic, epigenetic, phenotypic, and microenvironmental factors jointly shape evolutionary trajectories. These models highlight the role of non-genetic plasticity, spatial structure, and context-dependent selection in driving adaptation, resilience, and therapeutic resistance 11-13. Within this ecosystem view, tumor evolution is not solely the result of clonal competition driven by irreversible mutations, but also of reversible cell states, niche construction, and multi-scale interactions that determine evolutionary fitness.

Our synthesis extends classical Darwinian models of tumor evolution by integrating phenotypic plasticity as an evolutionarily relevant process. Rather than viewing cancer stem cells as a fixed subpopulation, we propose that stemness can be dynamically acquired in response to microenvironmental stress, particularly hypoxia. This introduces a non-genetic layer of heritable fitness that operates alongside mutational selection, accelerating adaptation and contributing to relapse and metastasis.

## Genetics of Tumor Evolution

Genetics and mutations play a fundamental role in tumor evolution, as they are the main drivers of the transformation of a normal cell into a cancerous cell and its subsequent progression towards a malignant state.

Tumor evolution begins with the accumulation of mutations in cellular DNA. These mutations can result from errors during DNA replication, exposure to environmental carcinogens such as ultraviolet rays, chemicals or radiation, or due to defects in the cell's DNA repair mechanisms. Over time, these mutations can affect key genes that regulate cell growth, programmed cell death (apoptosis), differentiation, and senescence, among other cellular processes 2, 14.

Moreover, mutations in oncogenes and tumor suppressor genes are particularly relevant in tumor evolution. Oncogenes are genes that promote cell growth and proliferation when activated, while tumor suppressor genes inhibit cell growth and promote cell death when functioning properly. Mutations that activate oncogenes (such as mutant *RAS* or upregulated *MYC*) or inactivate tumor suppressor genes (e.g., mutant *TP53,* silenced* PTEN*) can confer selective advantages on tumor cells, allowing them to proliferate uncontrollably and resist the body's cancer-suppressive mechanisms 15. TP53 (p53), for example, acts as a "guardian of the genome"; TP53 mutations (very common in cancer) lead to genomic instability, allowing the accumulation of further mutations and accelerating tumor progression toward more aggressive states 16.

In addition to point mutations in specific genes, tumor evolution also involves broader changes in the genome, such as the amplification or deletion of chromosomal regions, the rearrangement of chromosomes and the appearance of chromosomal abnormalities. Large-scale genomic changes have been identified in detail so far in thousands of tumors thanks to next-generation sequencing. These genomic alterations can affect gene expression, epigenetic regulation, and genome stability, thereby contributing to a great degree to tumor heterogeneity and plasticity. Although genomic alterations frequencies depend on the formation rate and the selective effects that could be conferred by each of them, it seems that, above all, complex rearrangement events may be key tumor drivers 17. Phenomena such as chromothripsis (massive chromosome breakage and reassembly) or chromosome duplication can reorganize the genome, alter gene expression, and generate new tumor properties without changes in the DNA sequence 18.

Classic studies by Vogelstein and Fearon proposed the *Multistage model* showing that colorectal cancer progresses by an ordered accumulation of genetic alterations, (e.g., APC, KRAS, TP53), demonstrating that successive mutations transform a benign polyp into invasive carcinoma. This directly illustrates how mutations direct the normal-to-cancer transition 19. Many works of massive sequencing (e.g., 20) describe the *genomic landscape* of cancers: few recurrent mutations that drive cancer (“drivers”) versus many neutral mutations (“passengers”), and how the identification of drivers explains cellular transformation and offers therapeutic targets. The TCGA Pan-Cancer project compared thousands of tumors and showed that, although tumors differ by tissue of origin, there are recurrently altered genetic pathways (e.g., PI3K, p53, RTK/RAS) that guide tumorigenesis and progression. This confirms on a large scale that genetics drives tumor evolution. Recent studies (e.g., 21) document clonal heterogeneity within a single tumor: genetically distinct subclones emerge through mutation and selection, this explains progression, metastasis, and the emergence of treatment resistance. The BCR-ABL gene fusion is a unique driver in most CML; The targeted inhibitor imatinib demonstrated that blocking this gene product can reverse the disease showing direct evidence that a specific genetic alteration drives tumorigenesis and is a therapeutic target 22, 23.

With all of this considered, both the accumulation of mutations and genomic alterations leads to the appearance of cellular subclones within a tumor, each with different genetic and phenotypic profiles. This clonal diversity is a central feature of tumor evolution and may arise due to the emergence of new mutations, the selection of beneficial genetic variants, or the migration of tumor cells to different anatomical sites. In summary, genetics and mutations play a crucial role in tumor evolution, providing tumor cells with the genetic adaptations necessary to survive, proliferate and spread in the body. Because these processes operate across time and space, tumor cells in different regions or at different times may carry distinct genetic profiles.

## Non-Genetic Tumor Evolution

Tumor evolution can occur even without the presence of genetic mutations. Although mutations are an important factor in the evolution of cancer, there are other mechanisms that can contribute to the adaptation and resistance of cancer cells without necessarily involving changes in DNA 24. Epigenetic changes, genome reorganization, selection of pre-existing variants, and interactions with the microenvironment may all contribute to the adaptation and resistance of cancer cells, allowing their evolution in the absence of additional DNA mutations 24. Non-genetic tumor evolution encompasses: epigenetic and transcriptional plasticity, regulation by non-coding RNAs, chromosomal reorganization, adaptation to the microenvironment, reversible states of dormancy or senescence. These mechanisms allow cancer cells to adapt, survive, and progress without any changes in their DNA sequence.

Stochastic aberrations in the epigenome, as well as in the genome, result also in intratumoral diversity. Changes in DNA methylation, histone modification, and other epigenetic processes can alter gene expression and cellular function without changing the DNA sequence. These changes can lead to the activation or deactivation of genes associated with proliferation, survival and resistance to treatment in cancer cells. For instance, dysregulation of epigenetic processes involved in developmental signaling pathways contributes to estrogen-receptor (ER^+^) breast cancer progression and endocrine therapy resistance 25**.** Also, for example, using single-cell multi-omics technologies, it has been shown that tumors can evolve through reversible changes in chromatin accessibility and gene expression programs, without the need for new genetic mutations.

Changes in expression of regulatory non-coding RNAs could vary the expression and activity of regulated target genes, therefore driving tumor evolution without genetic changes in the coding sequence 17, 26**.** MicroRNAs and lncRNAs can modulate the expression of key genes for proliferation, invasion, and drug resistance. These changes are heritable but reversible, and occur without altering the DNA sequence. Furthermore**,** genome reorganization and structural evolution without point mutations can exist.

The tumor microenvironment, including inflammation, hypoxia, and immune signals, exerts selective pressure that favors the survival of cells with adaptive characteristics 21, 27, 28. This evolution occurs through phenotypic selection rather than mutation 15. After exposure to chemotherapy or radiation, some cancer cells enter states of reversible senescence or dormancy. These states do not depend on mutations, but allow for survival and subsequent tumor relapse. Within a tumor, there may be pre-existing genetic and phenotypic heterogeneity, with subpopulations of cells that have different characteristics. Under the pressure of treatment or the tumor microenvironment, cells with certain favorable characteristics can be selected to survive and proliferate, leading to tumor evolution without the need for additional mutations. So much so that interactions with the tumor microenvironment, which includes factors such as vascularization, inflammation and immune response, can exert selective pressure on cancer cells and promote tumor evolution. For example, cancer cells can adapt to evade the immune response, undergo senescence or use pro-inflammatory signals to promote their growth and survival 15, 29-31. Immune pressure can induce changes in antigen expression, cytokine secretion, and metabolic pathways that allow tumor cells to evade the immune response without requiring new genetic alterations 32.

## Implications for Clinical Oncology

The existence of genetic and phenotypic heterogeneity has profound implications for clinical oncology. Because of spatial heterogeneity, a single biopsy may not capture the full diversity of a tumour. A sampled subclone may not represent the subpopulation that drives relapse or metastasis. Liquid biopsies (ctDNA) or multi-region sampling may offer better overview 33. Heterogeneity means that even if most tumour cells are sensitive to a therapy, resistant subclones may pre-exist (genetic heterogeneity) or may adapt via phenotypic plasticity (phenotypic heterogeneity) and survive. After selective treatment, these clones expand. For example, in EGFR-mutant NSCLC, resistance via secondary mutation or phenotypic transformation occurs 34.

Because of branching subclonal evolution and phenotypic plasticity, monotherapy targeting one driver may fail. Combination therapy targeting multiple pathways, or adaptive therapy that anticipates subclone emergence, may be required 35. Heterogeneity undermines biomarker reliability: a biomarker measured in one region may not reflect other tumour regions. Phenotypic heterogeneity further complicates prognosis because different subpopulations may have distinct metastatic potential, proliferative rates or drug responses 36, 37, highlighting the importance of personalized oncology.

Exposure to treatments such as chemotherapy, radiotherapy or targeted therapy can select subpopulations of tumor cells with different phenotypic characteristics. To survive stress created by anti-cancer therapies, tumor cells implement intrinsic or adaptive drug resistance mechanisms. This may lead to the emergence of therapy-resistant subclones and further contribute to tumor phenotypic heterogeneity 38.

Clinical translation challenges remain very present. Cost and complexity of multi-region/single-cell sampling, interpretation of massive data, translating heterogeneity measures into actionable therapy decisions, and ethical/logistical issues of repeated biopsies. Furthermore, we still need to identify which subclones matter most clinically (drivers of relapse/metastasis) vs which are “passengers”.

Genetic and phenotypic heterogeneity are fundamental facets of tumor evolution. While genetic heterogeneity has long been appreciated, phenotypic heterogeneity—driven by epigenetics, plasticity and microenvironmental adaptation—is now recognized as equally important in determining tumour behaviour, therapy response, progression and metastasis. Clinical examples in breast cancer, NSCLC, colon cancer and thyroid carcinoma illustrate how heterogeneity complicates diagnostics, prognostics and therapy. To effectively manage cancer, future strategies must incorporate an understanding of heterogeneity: using advanced single-cell/spatial methods, monitoring subclonal dynamics, designing therapies to target multiple subclones and phenotypes, and modulating the microenvironment (Table 1). Only by embracing the complexity of tumour heterogeneity can precision oncology realize its promise.

Recent large-scale analyses and integrative reviews underscore that tumor heterogeneity arises from combined genetic, epigenetic, transcriptional, and phenotypic sources, which collectively shape evolutionary potential 39, 40. These layers of heterogeneity interact dynamically**,** enabling rapid adaptation even in the absence of new genetic alterations.

## Clonal Diversity

Clonal diversity is a crucial aspect of tumor evolution and refers to the presence of multiple subpopulations of tumor cells within a tumor. This diversity arises due to the accumulation of genetic mutations and other molecular changes over time, as well as the selective pressures present in the tumor microenvironment.

As seen before, clonal diversity begins with the appearance of mutations in normal cells that give rise to the beginning of the tumor process, conferring selective growth advantage on some cells. These mutations can be spontaneous or induced by environmental factors such as exposure to carcinogens. As tumor cells replicate, more driver mutations can accumulate, generating cell subclones with different genetic and phenotypic profiles 41, 42.

In the tumor microenvironment, different subpopulations of tumor cells compete with each other for resources, such as oxygen and nutrients, as well as space to grow, having clonal selection. Furthermore, these cells are also subject to selective pressure exerted by the immune system and anticancer therapies. The interaction between circulating tumor cells (CTC) and platelets is a case in point of how the immune system can confer cell plasticity to tumor cells by inducing the acquisition of mesenchymal phenotypes and stemness features. As a result, cells with mutations that confer proliferative advantages, resistance to programmed cell death (apoptosis), or resistance to therapy can be selected for survival, expansion and metastasis 43.

Clonal diversity is manifested in the genetic and phenotypic heterogeneity observed within a tumor. Cell subclones may differ in terms of genetic mutations, gene expression, proliferative capacity, drug resistance, and other traits. This heterogeneity can complicate cancer diagnosis and treatment, as some subclones may be more aggressive or resistant to therapy than others 44, 45. For instance, the poor response and the rapid relapse after first-line treatment in small cell lung cancer (SCLC) seem to be caused by a treatment-dependent increase in clonal diversity and clones' evolutionary adaptation after treatment-induced pressure 46, 47. We'll explore this point more in detail below.

Advances in spatially resolved transcriptomics and proteomics have revealed that tumor evolution is profoundly shaped by spatial organization and localized microenvironmental pressures. Recent studies demonstrate region-specific evolutionary trajectories and reciprocal tumor-stroma interactions that influence immune evasion, invasion, and therapy response 48, 49.

Clonal diversity has important clinical implications for cancer management. For example, detection of clonal diversity using next-generation sequencing techniques and biomarker analysis have illuminated different aspects of the pathogenesis, maintenance and progression of myeloid malignances 50.

## Genetic and Phenotypic Heterogeneity

Tumor evolution is fundamentally shaped by heterogeneity in both genetic (differences in DNA sequence or structure among cancer cells) and phenotypic (differences in observable cell behaviour, morphology, expression, metabolism, etc.). Heterogeneity allows cancer cell populations to adapt, survive selective pressures (such as therapy, microenvironmental stress), and gives rise to progression, metastasis and treatment resistance.

As seen so far, genetic heterogeneity arises from the accumulation of somatic mutations, copy number alterations (CNAs), structural rearrangements, chromosomal instability (CIN) and other genomic and non-genomic processes (ncRNA, methylation, etc). These changes generate subclonal populations within a tumor, each harboring different driver or passenger alterations. The classical model of clonal evolution defends that rare cells acquire advantageous mutations, expand clonally, and over time further subclones arise under selection 3. From a population genetics perspective, intra-tumor heterogeneity (ITH) represents a cancer cell population in which subclones compete, drift, proliferate or are eliminated 21, 51.

Large-scale sequencing studies (e.g., multi-region sequencing) have shown that many solid tumors have branching evolutionary patterns rather than linear. For example, in 52, 53 the authors highlight that spatial heterogeneity in both genotype and phenotype complicates precision oncology. In breast cancer, intra-tumour heterogeneity is well documented at genetic, transcriptomic, and microenvironmental levels 36, 37**.** In a given tumour mass, different regions may harbor different PIK3CA mutations, variable copy number changes of HER2, variable TP53 mutations or independent subclonal expansions. Such heterogeneity means that a single biopsy may not represent the full diversity of the tumor, and may miss subclones that drive therapy resistance or metastasis.

Genetic heterogeneity fosters resistance because distinct subclones may already carry or acquire resistance-conferring mutations 54. Under therapy, sensitive clones are eliminated, but resistant subclones expand. In non-small cell lung cancer (NSCLC) with EGFR mutation, for example, subclonal EGFR T790M or MET amplification may pre-exist, accounting for resistance to EGFR-TKIs 55-57.

Changes in gene expression that do not involve alterations in the DNA sequence, *epigenetic changes*, can significantly contribute to phenotypic heterogeneity. These changes can include modifications in DNA methylation, histone acetylation, and microRNA regulation, which affect gene activity and can lead to different cellular phenotypes within a tumor. Consequently, among the phenotypic diversity, the fittest cells are selected for malignant progression 58.

All these changes reflect on* t*umor cells exhibiting phenotypic plasticity, meaning that they have the ability to change their state and characteristics in response to signals from the environment, including alterations in nutrient availability, immune response, hypoxia or therapy, among others. This can result in a wide range of phenotypes observed within the tumor and between primary tumor and metastases, making it difficult to identify a single cell population as a therapeutic target 59, 60.

The environment surrounding tumor cells, known as the tumor microenvironment, plays a crucial role in cancer cells plasticity, especially CSC, therefore regulating their phenotypic heterogeneity 28. Tumour cell populations adapt not just via genotype but via interaction with their microenvironment (oxygen level, stromal cells, immune milieu). This adaptation creates functional heterogeneity and influences evolution. This interplay between genotype and phenotype marks the way in tumour evolution. Factors such as oxygen availability (hypoxia), the presence of immune cells, poor vascularization and cellular communication through chemical signals can influence the response of tumor cells and their phenotypic behavior. Moreover, multiple niches can coexist in one tumor increasing the cellular diversity 61, 62.

### Phenotypic Heterogeneity

While genetic heterogeneity is central, phenotypic heterogeneity, differences in behavior, morphology, gene expression, metabolism, differentiation state, microenvironmental interactions, may be just as critical. Indeed, cells with similar or even identical genotypes may display markedly different phenotypes due to epigenetic states, microenvironmental context, stochastic gene expression, cell cycle states, and plasticity.

Lenz and colleagues 63 explored how, even among cells with similar genomes, variability in the epigenome, transcriptome, proteome, metabolome and signaling network generates major phenotypic diversity, and 7, 64 emphasized that non-genetic ITH, including epigenetic, metabolic, microenvironmental influences, is a major barrier to therapy and metastasis. Also, Teeuwssen and Fodde 65 describe how metastasis in colon cancer is underlined not only by accumulation of genetic alterations (sequence from adenoma to carcinoma) but also by phenotypic plasticity, cells adopt transient and reversible states (e.g., epithelial-to-mesenchymal transition, stemness) to adapt to new microenvironments. Thus, phenotypic heterogeneity enables adaptation and metastasis even in the absence of new driver mutations.

Phenotypic states such as dormancy, quiescence, epithelial-mesenchymal transition (EMT), cancer stem cell-like behaviour or metabolic adaptation allow subpopulations of tumour cells to survive therapy. These states may be reversible and may not require new mutations. For instance, in NSCLC, phenotypic transition from epithelial to mesenchymal phenotype (loss of E-cadherin, gain of vimentin) is associated with resistance to EGFR inhibitors 66-68. In the work on 69 the authors note that epigenetic dysregulation occurs more frequently than gene mutations and contributes substantially to intratumor phenotypic heterogeneity.

### Interplay of Genetic and Phenotypic Heterogeneity

In reality, tumor heterogeneity is shaped by the interplay of genetic and phenotypic layers. Genetic variation sets the potential for subclone diversity; phenotypic heterogeneity determines how those subclones behave in the microenvironment, respond to stress or therapy, and adapt. In other words: genotype frames possibility; phenotype determines actual behaviour. The fate of individual tumor subclones depends not only on their genomic profile but also on how well that profile and the tumour microenvironment (TME) phenotype harmonise—i.e., the cancer cell mutations shape the TME, and in turn the TME influences which clones survive 70, 71.

Spatial heterogeneity further accentuates this interplay, different regions of a tumour may have different oxygenation, nutrient supply, immune infiltration, stromal context, etc., so that subclones with distinct genotypes may thrive in one niche and not in another, and cells with the same genotype may behave differently due to microenvironmental differences.

Clinical multi-region sequencing efforts have shown that while many early driver mutations are ubiquitous (clonal) across a tumour, later subclonal mutations are region-specific. However, phenotypic heterogeneity (e.g., expression variability, microenvironmental adaptation) may be even more spatially diverse. For example, in hepatocellular carcinoma (HCC) RNA-based phenotypic heterogeneity evolves “decoupled” from genetic heterogeneity: different phenotypic subtypes (RNA/immune) may co-exist and evolve in different regions though the genomic differences are minor 70-72. Thus, genetic heterogeneity creates the substrate; phenotypic heterogeneity and plasticity determine functional diversity and adaptation; together they drive tumor evolution, metastasis, and resistance.

In metastatic breast cancer, studies comparing primary tumour, metastasis and circulating tumour DNA (ctDNA) demonstrate high genetic heterogeneity and complex clonal evolution. Kavan and colleagues 73 report how differently evolving genetic clones drive progression and clinical outcome. The authors show that the “linear progression” model is less frequent than expected; instead, parallel or branched evolution is common, and ctDNA often more closely reflects the metastasis than the primary tumour. This has clear clinical consequences: a biopsy of primary tumour may miss subclones responsible for relapse or metastasis; liquid biopsy may capture evolving clones and help guide therapy. Furthermore, phenotypic heterogeneity is evident: different subclones may express distinct hormone-receptor status, have differential proliferative/ invasive capacities, or respond differently to therapy. Thus, precision therapy must contend with subclone diversity.

In NSCLC harbouring EGFR mutations, initial responses to EGFR-TKIs are often positive. However, resistance eventually arises. Resistance mechanisms include secondary mutations (e.g., T790M), MET amplification, and also phenotypic transformation (e.g., small cell lung cancer (SCLC) transformation, or EMT). The work 55 described how plasticity and heterogeneity complicate therapy. Clinically, about 5-15% of EGFR-mutant NSCLC transform to SCLC phenotype after EGFR-TKI therapy—a phenotypic shift without necessarily new driver mutations. The phenotypic heterogeneity (EMT, neuroendocrine differentiation) allows survival and progression. Thus, both genetic heterogeneity (multiple subclones) and phenotypic heterogeneity (plastic states) drive therapy failure.

In colon cancer, the adenoma-to-carcinoma sequence is genetically well-defined (from APC to KRAS to TP53). But metastasis formation involves phenotypic plasticity and heterogeneity 65. Disseminated tumour cells adapt via transient phenotypic states (EMT, stemness) to colonize distant sites. This indicates that even with a shared genotype, tumour cells must traverse multiple phenotypic states (motile, invasive, survival in circulation, colonization) to form metastases, and not all cells can do this. Heterogeneity in phenotype dictates which subpopulations succeed. Clinically, this suggests that targeting only the primary tumour genotype may not suffice; phenotypic plasticity must be addressed.

In breast, within a single tumour there may be subpopulations differing in gene expression (e.g., basal vs luminal features), proliferation rate, hypoxia adaptation and microenvironment interactions 36, 37. For example, triple negative breast cancer (TNBC) often shows high intratumour heterogeneity; different subclones may respond differently to chemotherapy, some entering quiescent states. This heterogeneity is a major obstacle to achieving durable remissions. One clinical study found that subclonal PIK3CA mutations predicted worse outcomes as they indicated genetic heterogeneity existing at diagnosis 33.

In thyroid carcinoma 74, the intratumour heterogeneity (genetic + phenotypic + microenvironmental) can affect the validity of biomarker testing in routine clinical practice. For example, an initial biopsy may sample a region with low grade tumour cells, but a separate region may harbour more aggressive clones with different phenotypes. This affects grading, prognostication and therapy choice.

## Clonal Heterogeneity in Tumor Evolution by Stochastic Variations

Clonal heterogeneity in tumor evolution can be influenced by stochastic variations in gene transcription. This refers to random variability in gene activity that can arise due to stochastic processes within cancer cells. The stochastic variations in transcription may contribute to clonal heterogeneity and cancer evolution in several ways:

*Fluctuating gene expression*: Gene activity can fluctuate randomly over time due to stochastic processes in the transcription and translation machinery. This can result in subpopulations of cells with different levels of gene expression, even within a single tumor.

*Production of RNA variants*: Stochastic variations in transcription can lead to the production of different messenger RNA (mRNA) variants from the same gene. These variants may encode proteins with different functions or may regulate the expression of other genes differently, contributing to functional heterogeneity within the tumor.

*Random gene regulation*: Gene regulatory processes, such as transcription factor binding and chromatin modification, can be subject to stochastic fluctuations. This can affect gene activity in different cells in unpredictable ways, contributing to heterogeneity in response to environmental signals and therapies.

*Adaptation to the microenvironment*: Cancer cells can face variable conditions in their environment, such as changes in the availability of oxygen, nutrients, and signaling signals. Stochastic variability in gene transcription may allow some cells to better adapt to these changing conditions, contributing to heterogeneity in response to the tumor microenvironment.

## Darwinian Evolution and Cancer

Charles Darwin's theory of evolution is based on the idea that species evolve over time through a process of natural selection. This process implies that amongst all organisms, those showing preexisting favorable characteristics for surviving and reproducing in their specific environment are more likely to transmit those characteristics to subsequent generations. There are several types of Darwinian evolution that can be observed in nature (Table 2):

*Divergent evolution.* This type of evolution occurs when two or more species share a common ancestor, but over time they become increasingly different due to natural selection acting in different directions due to distinct environmental conditions. The Galapagos finches studied by Darwin, which have different adaptations according to the different islands on which they live 75, and the speciation of dogs from domestication of wolves 76 constitute classic examples of divergent evolution.

In tumor evolution, cancer cells can arise from a single progenitor cell but become different over time due to genetic mutations and epigenetic changes, giving rise to subpopulations of cells with distinct characteristics, such as drug resistance or invasive capacity 77. For example, studies by Navin et al. have demonstrated that different clones in breast tumors with distinct genomic profiles arise from a common ancestor 78, 79.

Multiregion exome sequencing of primary clear-cell renal cell carcinomas and matched metastases revealed *branched* evolutionary trees showed that many somatic mutations were present only in some regions (subclonal), while different regions carried distinct inactivating mutations in the same tumor-suppressor genes (convergent functional evolution). This demonstrates that a single biopsy can miss clinically relevant subclones 80.

Also, whole-genome sequencing of multiple metastases from men who died of prostate cancer reconstructed evolutionary histories showing *branched divergence*, polyclonal seeding of metastases, and metastasis-to-metastasis spread (reseeding). Clinically, this explains how different metastatic sites can carry distinct actionable alterations and why resistance/relapse may reflect divergent subclones 81.

TRACERx trial in non-small-cell lung cancer prospectively sampled multiple regions per tumour and showed frequent spatial and temporal *branched* subclonal diversification, where early (truncal) drivers were ubiquitous but many clinically important events (e.g., HLA loss, subclonal drivers) were branch-specific. The work links subclonal diversity to outcome and immune escape, stressing that treatment guided by a single sample can miss subclonal resistance mechanisms 82.

In glioblastoma, divergent transcriptional and genetic states at single-cell resolution was found. Single-cell RNA-seq of primary glioblastoma revealed coexisting malignant cell subpopulations with divergent transcriptional programs (proliferative, mesenchymal-like, neuronal-like etc.), often aligned with different genetic alterations in subclones. This intratumour divergence contributes to variable response to therapy and rapid relapse in glioblastoma 83.

*Convergent evolution*. Unlike divergence, convergent evolution occurs when unrelated species evolve similar characteristics due to similar selective pressures in their environments. This can happen when species face similar environmental challenges and evolve similar solutions independently. A common example is the evolution of wings in bats, birds, and insects, which allows them to fly even though they do not share a common ancestor with flight characteristics. C*onvergent evolution* in cancer refers to distinct tumor subclones independently acquiring different genetic or epigenetic alterations that produce the same functional or phenotypic outcome (e.g., activating the same oncogenic pathway by different mutations). In tumor evolution, different tumors and cells of the tumor microenvironment can acquire similar characteristics, such as resistance to treatments, despite having different genetic origins, due to similar selective pressures of the tumor microenvironment, such as nutrient availability or immune pressure. For example, different tumor microenvironment cells show convergent characteristics despite their various origins due to the activation of *MYC* in the tumor cells in a murine model of prostate cancer 84.

Convergent Loss of Chromatin-Remodeling Genes in Clear-cell Renal Cell Carcinoma (ccRCC). Multiregion sequencing revealed that spatially distinct regions of the same renal tumor had different mutations in the same tumor suppressor genes (e.g., *SETD2*, *PTEN*, *KDM5C*), each leading to loss of gene function. This is a hallmark example of convergent evolution, where separate branches of the tumor tree independently achieve the same functional inactivation. Explains how single biopsies can miss therapeutically relevant mutations; underscores redundancy in tumor evolution 85.

Convergent Activation of the Androgen Receptor (AR) Pathway in Metastases in Prostate cancer. Sequencing of multiple metastatic sites from lethal castration-resistant prostate cancer showed different mechanisms (AR gene amplification, point mutations, enhancer duplication, or splice-variant expression) that all reactivate the AR pathway, a textbook example of *convergent evolution* toward endocrine resistance. Different metastases can evolve distinct mechanisms to restore the same oncogenic signaling, complicating therapy with AR antagonists 86. Integrated genomic analyses also revealed that within the same GBM tumor, different subclones harbored distinct amplifications or mutations in RTK genes (*EGFR*, *PDGFRA*, *MET*), all converging to activate the PI3K/MAPK signaling axis 87. Therapies targeting a single RTK may fail because different subclones activate the same downstream pathway through alternative routes.

In NSCLC, *EGFR*-mutant tumors treated with EGFR inhibitors often develop different secondary mutations or bypass alterations (e.g., *MET* amplification, *HER2* amplification, *KRAS* activation), independent mechanisms that converge to restore signaling downstream of EGFR. Demonstrates convergent molecular adaptation under drug pressure; multiple resistance routes lead to the same resistant phenotype 88 89. In patients with metastatic colorectal cancer treated with anti-EGFR antibodies, independent emergence of KRAS, NRAS, or EGFR extracellular-domain mutations occurs in different metastatic sites or circulating subclones, all leading to resistance to EGFR blockade. Represents convergent functional adaptation — multiple independent mutations achieving the same therapy-resistant phenotype 90 91. On other example, Melanoma patients treated with BRAF inhibitors (vemurafenib/dabrafenib) develop multiple independent resistance mechanisms (such as *NRAS* mutations, *MEK1/2* mutations, *BRAF* splice variants, or *BRAF* amplification) all of which restore MAPK signaling 92**.** This convergent evolution of resistance mechanisms explains why targeting one node (BRAF) is insufficient and underlies combination therapy strategies (e.g., BRAF + MEK inhibitors).

Spatially resolved sequencing of PDACs has revealed distinct subclones harboring different mutations in KRAS or its downstream effectors (e.g., *BRAF*, *MAP2K1*), each providing equivalent pathway activation. This convergent activation underlines the dominance of MAPK signaling in pancreatic tumor evolution 93. Virtually all SCLC cases show biallelic inactivation of TP53 and RB1, achieved by different combinations of point mutations, deletions, or loss of heterozygosity, a good example of convergent evolution at the tumor-type level 94. Finally, Comparative sequencing of primary and recurrent glioblastoma samples demonstrated different subclones acquiring distinct mutations (e.g., *PTEN*, *NF1*, or *PIK3CA*) that all lead to PI3K/AKT pathway activation under treatment pressure 95.

Furthermore, divergent and convergent evolution was found in metastatic lesions. Comparative studies of matched primary tumours and multiple metastatic sites report *divergent evolution* of metastases (different sites harbor site-specific driver events) and in some cases *convergent evolution* (independent acquisition of similar functional changes). Clinically this suggests metastasis-directed therapy should consider site-specific molecular profiles, because different metastases can diverge genetically and phenotypically 96.

While the classical model of tumour evolution emphasizes a branching process leading to spatial and temporal heterogeneity, a growing body of evidence demonstrates that evolution within a tumour is often convergent, reflecting the fact that tumour cells are subject to shared constraints and selective forces that favour certain advantageous phenotypes. Many of these convergent phenotypes map directly onto the Hallmarks of Cancer, as described by 2, 15, 58, which represent recurrent functional capabilities that promote malignant growth across cancer types. Examining convergent evolution through the lens of the hallmarks reveals how similar pressures repeatedly drive tumour cells toward comparable adaptive outcomes.

Convergent evolution occurs when different cancer cell lineages independently acquire the same or functionally similar mutations. These changes need not involve identical DNA substitutions; rather, the resulting phenotypes are what converge. For instance, metastases from the same primary tumour frequently evolve distinct mutations affecting the same pathway—such as different activating mutations in *PIK3CA* or loss-of-function lesions in *TP53*—reflecting the selective advantage associated with disruption of these nodes 1, 4, 85.

This recurrent acquisition of similar changes is consistent with the idea that tumour cells face predictable selective pressures. These include nutrient deprivation, immune surveillance, spatial constraints, hypoxia, and—when treated—drug exposure. As a result, evolution repeatedly drives cells toward known solutions, many of which are encapsulated in the hallmarks framework. In this view, the hallmarks can be understood as attractor states in the evolutionary landscape of tumour progression.

Several biological and ecological factors explain why parallel evolution is prevalent: Even though tumours are heterogeneous, certain stressors—hypoxia, nutrient limitation, immune activity—are present across many niches. These common pressures drive subclones toward similar adaptive solutions. The number of pathways capable of sustaining hallmark phenotypes is finite. For example, only a handful of nodes robustly amplify proliferation or disable apoptosis. This funneling effect encourages convergence. Tumours, especially those with DNA repair defects, generate mutations at an accelerated pace, increasing the likelihood that distinct subclones will stumble upon similar adaptive mutations. Physically separated tumour regions—different lobes of a tumour or distant metastases—evolve largely independently, creating multiple opportunities for similar solutions to emerge. Finally, targeted therapies and immunotherapies impose extreme selection pressures. Tumours respond with parallel resistance mechanisms, providing some of the clearest real-time examples of convergent evolution.

*Parallel evolution*. This type of evolution occurs when two related but separate species evolve in similar directions after having separated from a common ancestor. Although species may be found in different geographic locations, they may face similar selective pressures, leading to the evolution of similar traits. An example is the evolution of zebras and horses, which evolved independently but have similar physical characteristics due to their similar habitats and adaptations to escape predators. *Parallel evolution* in cancer means independent emergence of the same or functionally similar genetic/phenotypic changes in different subclones or tumour sites (e.g., the same gene mutated independently in separate tumour regions or metastases). In tumor evolution, different tumors can evolve independently but develop similar characteristics due to similar selective pressures in the tumor microenvironment.

In clear-cell renal cell carcinoma, independent inactivation of the same tumour suppressors across regions. Multiregion exome sequencing of primary clear-cell renal cell carcinomas showed *branched evolution* and parallel (independent) inactivation of the same tumour-suppressor genes (e.g., SETD2, PTEN, KDM5C) in different regions of the same tumour. This demonstrates that functionally equivalent hits can arise independently in separate subclones — with clear implications for sampling bias and targeted therapy 85. In Colorectal cancer or lung tumors, independent (parallel) emergence of KRAS mutations causing anti-EGFR resistance. In patients treated with anti-EGFR antibodies, multiple independent KRAS mutations (different codons/alleles) emerged in distinct metastases or in circulating DNA, representing parallel evolution toward the same phenotypic outcome (EGFR-resistance). This shows that separate subclones can converge on the same resistance mechanism by independently acquiring KRAS mutations 90, 97.

The TRACERx multi-region study of NSCLC found many branch-specific alterations and parallel events such as independent loss-of-heterozygosity (LOH) at HLA alleles and repeated selection of functionally similar subclonal changes, indicating that distinct subclones can independently evolve immune-escape or other fitness traits. Clinically relevant because it links subclonal diversity to outcome and immune evasion 35, 82.

Whole-genome sequencing of multiple metastases in lethal prostate cancer reconstructed branched histories with polyclonal seeding and independent (parallel) acquisition of alterations in different metastases — showing that different metastatic sites may independently evolve similar or distinct driver alterations (important for therapy selection across sites) 81.

Treatment-monitoring (plasma/ctDNA) studies found independent emergence of multiple resistance mutations (parallel evolution under therapy). Serial plasma (ctDNA) sequencing in patients receiving targeted therapy often reveals multiple, independent resistance mutations arising over time (e.g., independent EGFR T790M alleles, PIK3CA, RB1, etc.), indicating parallel selection of resistance strategies by separate subclones. This has clinical impact for non-invasive monitoring and choosing subsequent lines of therapy 98, 99.

Several pathology and genomics reviews compile examples where parallel evolution (independent but similar genomic changes in separate subclones) mimics inter-tumour diversity and has diagnostic/therapeutic consequences (sampling bias, convergent resistance). These reviews synthesize multiple clinical datasets showing the phenomenon across cancer types 80. Single-cell sequencing studies in breast cancer and glioblastoma revealed multiple distinct clonal branches within the same tumour; in several cases different subclones independently display functionally similar programs (e.g., proliferation, invasion, therapy-resistant transcriptional states), a cellular-level form of parallel evolution relevant for therapy resistance and metastasis 83, 100.

In each case, separate subclones or tumour sites independently acquired similar functional changes (same gene mutated independently, same pathway altered, or similar phenotype), not by descent from a single recent common mutant, but by independent events selected because they confer similar fitness advantages (e.g., drug resistance, immune escape, growth). Clinically this matters because: (1) single biopsies can miss independently evolved actionable/resistance events; (2) different metastases can require different therapeutic approaches; (3) monitoring by ctDNA can capture parallel resistance events evolving in separate subclones.

Importantly, distinguishing among evolutionary modes requires careful consideration of evidentiary standards. Convergent evolution is supported when distinct genetic alterations in separate lineages independently affect the same pathway or functional outcome, whereas parallel evolution requires the repeated acquisition of identical or highly similar genetic events in independent clones. Both interpretations rely on robust phylogenetic reconstruction and multi-region or longitudinal sampling to demonstrate independent origins. In contrast, phenotypic convergence, such as acquisition of stem-like or drug-tolerant states, may occur without shared genetic drivers and instead reflects selection on reversible cell states. Discriminating among these scenarios therefore necessitates integration of genetic, epigenetic, transcriptional, and spatial data.

*Coevolutionary evolution*. This type of evolution occurs when two or more species interact closely with each other and each exerts selective pressure on the other. This may result in reciprocal evolutionary changes in both species over time. A classic example is coevolution between plants and pollinators, where plants develop specific characteristics to attract certain pollinators, and in turn, pollinators can develop adaptations to better exploit plant resources. In tumor evolution, tumor cells can interact with and exert selective pressure on the surrounding microenvironment, which in turn can influence tumor evolution and its ability to grow, invade, and spread.

1. to species in nature, tumor cells in the human body are subject to selective pressures, and their evolution over time is also determined by the hostile environment in which they develop. Although it was not known in Darwin's time, the mechanisms that rule both species and tumor evolution are the aforementioned genetic and non-genetic factors, which contribute to phenotypic diversity in nature and clonal heterogeneity within a tumor. In fact, the models of Darwinian evolution observed in nature can be compared to those that govern the evolution of a tumor.

Multi-region profiling of early, treatment-naïve NSCLC revealed spatially heterogeneous immune infiltration and ongoing immunoediting, tumor regions with high immune infiltration showed loss of neoantigens (by HLA loss or promoter hypermethylation), while immune-cold regions retained neoantigens. This documents tumor and immune co-evolution during natural disease and links immune selection to worse disease-free survival 101. Allele-specific HLA loss and immune escape in lung cancer (immune selection driving HLA LOH). Tumors under immune pressure lose specific HLA alleles (loss-of-heterozygosity) in subclones, permitting expansion of those subclones by evading T-cell recognition, a direct example of selection by the immune system shaping tumor genotype and clonal architecture 51, 102.

In melanoma patients treated with anti-PD-1 (nivolumab), responders frequently showed decreases in tumor neoantigen burden and expansion of T-cell clones — evidence that immunotherapy can drive selection against neoantigenic subclones (therapy-mediated co-evolution of tumor and immune repertoire). This links genomic changes in the tumor to evolving immune responses on treatment 103.

Longitudinal single-cell and genome profiling in multiple myeloma patients documented parallel changes in malignant plasma-cell subpopulations and the immune compartment across disease stages, mapping a co-evolutionary trajectory that helps explain progression and relapse 104.

Pancreatic ductal adenocarcinoma (PDAC) progression is driven by tight reciprocal signalling between KRAS-mutant epithelium and cancer-associated fibroblasts (CAF)/stellate cells represents Epithelial /stromal co-evolution. In PDAC, the epithelium activates stromal cells (e.g., via SHH/TGFβ), which in turn remodel matrix, secrete growth/metabolic support, and shape immune infiltration. This evidence that the tumor and stroma evolve together, creating therapy-resistant microenvironments 105.

Tumor/vasculature co-evolution leads to anti-angiogenic resistance (for example, colorectal liver metastases). Some liver metastases grow by co-opting pre-existing host blood vessels rather than by angiogenesis; such tumors are less responsive to anti-angiogenic therapy (e.g., bevacizumab). This demonstrates co-evolutionary adaptation of tumor cells to exploit a vascular microenvironmental strategy that undermines therapy 106.

Tumor/microbiome co-evolution. Clinical and experimental studies found enrichment of *F. nucleatum* in CRC tissues (and in patients with recurrence after chemotherapy). *F. nucleatum* interacts with tumor cells (E-cadherin/β-catenin signaling), modulates innate immunity (TLR4/MYD88) and autophagy, and can promote chemoresistance, an example of reciprocal interactions in which microbiota composition and tumor behavior shape each other and clinical outcome 107, 108.

Therapy-driven phenotypic co-evolution: histologic transformation under targeted therapy (EGFR-mutant NSCLC to SCLC). In patients on EGFR TKIs, some tumors transform histologically from adenocarcinoma to small-cell lung cancer (SCLC) as a mechanism of resistance. This is a co-evolutionary/adaptive phenotypic shift under drug selection where tumor cells change lineage/phenotype (and associated vulnerabilities), forcing a shift in clinical management. See multiple clinical series/reviews describing EGFR to SCLC transformation as an acquired resistance mechanism 109.

In all the examples above, the tumor does not evolve in isolation: selective pressures (immune cells, stromal cells/CAF, blood vessels, microbiota, or therapy) actively shape which tumor clones expand, and tumor cells reciprocally remodel those partners (e.g., by secreting signals, changing antigenicity, or recruiting microbes). Clinically these matters because co-evolution produces heterogeneous and therapy-adapted ecosystems, explaining immune escape, microenvironment-mediated resistance, failure of mono-targeted therapies, and changes in tumor phenotype that require altered treatment strategies.

Failure to meet these evidentiary standards risks over-interpreting adaptive evolution, with direct consequences for clinical decision-making, biomarker selection, and therapeutic targeting.

### Additional Evolutionary Trajectories in Tumor Progression

*Neutral Evolution and Genetic Drift.* Not all intratumour heterogeneity arises from positive selection. In many tumors, especially during early expansion, a substantial fraction of genetic diversity appears to accumulate through neutral evolution and genetic drift, where mutations confer neither selective advantage nor disadvantage. In this context, clonal frequencies are shaped primarily by stochastic processes rather than fitness differences.

Neutral evolution generates extensive genetic heterogeneity without clear driver selection, complicating the identification of actionable mutations and limiting the predictive value of single biopsies. Clinically, tumors dominated by neutral evolution may appear genetically diverse yet phenotypically stable, whereas selection-driven evolution often correlates more strongly with therapy resistance and aggressive behavior. Distinguishing neutral from selective dynamics therefore has direct implications for biomarker development, treatment stratification, and evolutionary forecasting.

Recent work has further refined our understanding of neutral evolution by demonstrating genome-level constraints and selection acting on mutational processes themselves, blurring the classical distinction between neutral drift and selection 110.

Modern phylogenetic reconstruction approaches integrate bulk, multi-region, single-cell, and longitudinal data to infer evolutionary histories with increasing resolution, while explicitly accounting for sampling bias and uncertainty 111.

*Punctuated (“Big Bang”) Tumor Evolution.* An alternative to gradualist models is punctuated or “Big Bang” evolution, in which extensive genetic diversification occurs early during tumor initiation, followed by predominantly neutral expansion. This model explains the presence of high intratumour heterogeneity with minimal ongoing selection and has been observed in several cancer types, including colorectal and breast cancer.

Multi-region profiling in colorectal tumours supported a “Big Bang” model in many cases: a burst of early diversification creates numerous intermixed subclones that persist and occupy different regions (early divergence rather than strict linear progression). Clinically, the model explains spatially divergent mutation patterns and why metastasis/therapy resistance may arise from early subclones not obvious in a single sample 112. Also, single-cell (single-nucleus) genomic sequencing in primary breast tumours uncovered *distinct clonal subpopulations* (different copy-number profiles/clonal branches) within the same tumour; some tumours showed punctuated clonal expansions while others showed multiple coexisting branches. Clinically, this explains heterogeneity of metastatic seeding and treatment response 78, 79.

Clinically, punctuated evolution implies that metastatic potential and therapy resistance may be encoded early in tumor development, limiting the effectiveness of late intervention strategies. It also challenges linear progression models and suggests that early detection and intervention may be critical, as later treatments may be acting on a pre-diversified and evolutionarily entrenched tumor ecosystem.

*Evolution of Reversible Cell States and Phenotypic Plasticity.* Beyond fixed genetic alterations, tumors evolve through reversible cell-state transitions, including drug-tolerant persistent states, epithelial-mesenchymal plasticity, and lineage switching. Although these states may be transient at the single-cell level, they are repeatedly selected under therapeutic and microenvironmental pressure, effectively functioning as bona fide evolutionary trajectories.

These reversible states provide a rapid, non-genetic route to adaptation, allowing tumors to survive acute stress and later re-expand. Clinically, this explains minimal residual disease, adaptive resistance, and relapse without new driver mutations. Targeting the regulatory networks underlying plasticity, rather than static genetic lesions, may therefore be essential for durable therapeutic responses.

Together, these evolutionary modes highlight that tumor progression is not governed by a single evolutionary logic, but by the dynamic interplay of genetic diversification, stochastic processes, and environmentally induced phenotypic plasticity, processes that collectively shape clinical outcome and therapeutic response (Table 2).

## Cellular Homeostasis and Tumor Evolution

**Figure 4:**
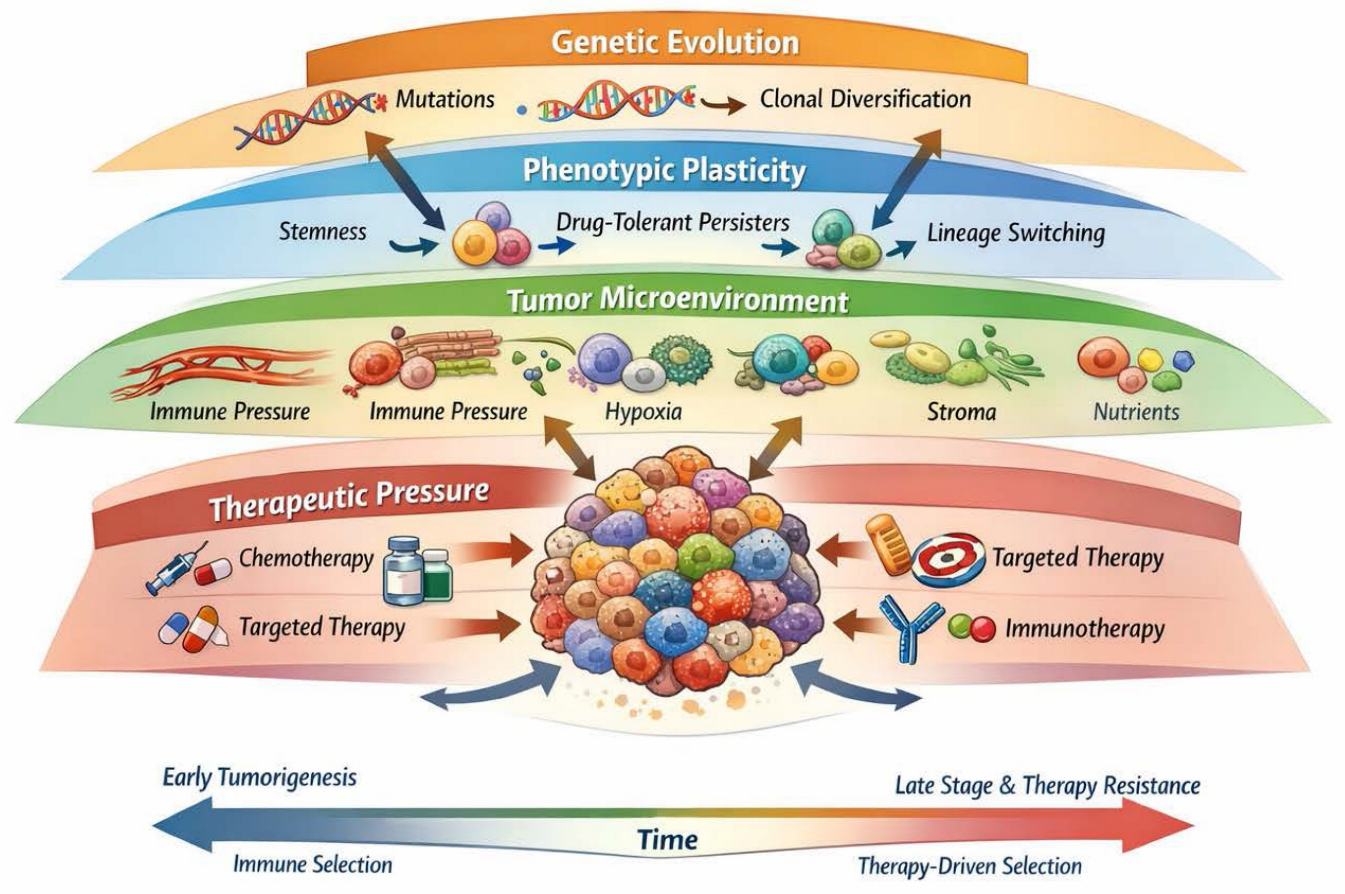
Tumors as dynamic evolutionary ecosystems*.* Tumor evolution emerges from the integration of genetic selection, reversible phenotypic states, and context-dependent microenvironmental and therapeutic pressures.

Tissue or cellular homeostasis refers to the dynamic balance that tissues and cells maintain to ensure their proper functioning within an organism (Figure 4). This balance involves the regulation of several factors, such as cell proliferation, programmed cell death (apoptosis), cell differentiation, and interaction with the surrounding microenvironment. In the context of tumour evolution, cancer cells challenge this normal cellular homeostasis in several ways: uncontrolled proliferation; resistance to apoptosis; changes in cell differentiation; and interaction with the tumour microenvironment. It is another way to explore similar trait to their evolution towards the hallmarks of cancer, to explore each of these components with mechanistic and clinical perspectives, and illustrate how disrupting cellular homeostasis underpins tumour initiation, progression, therapy resistance and metastasis.

Homeostasis is a hallmark of healthy multicellular life: cells proliferate when needed, differentiate as required, senesce or die when damaged, maintain tissue architecture and interact in regulated crosstalk with their microenvironment (stroma, immune cells, vasculature). The integrity of this system is essential for organ function and prevention of neoplastic transformation. When homeostatic controls fail, the risk of tumour development rises.

The evolution of tumours can be viewed as a process in which individual cells or sub-populations escape homeostatic regulation, gain competitive growth advantages, and adapt to microenvironmental and therapeutic pressures. According to this cancer theory, cancer is not simply uncontrolled proliferation alone but is a dynamic evolutionary process, transformed cells subject to mutation (genetic & epigenetic), selection, drift and adaptation.

### Mechanisms of Proliferation Deregulation

Normal cells decide whether to divide or remain quiescent based on extracellular signals (growth factors, nutrients, cell-cell contact), intracellular checkpoints (DNA damage, replication stress) and tissue-level cues. In many cancers, these regulatory circuits are disrupted: oncogenes become constitutively activated; tumour suppressor genes are inactivated; cell-cycle checkpoints fail; control of the G1/S and G2/M transitions is lost 113. Classic reviews show that deregulation of the cell cycle (for example Cyclin D1, CDK4/6, p16INK4a, Rb, p53) is nearly universal in human cancers 114. For instance, the G1 phase “restriction point” is often bypassed in tumour cells, allowing unscheduled entry into S-phase, replication of damaged DNA and accumulation of aberrations 115.

Another layer is signalling deregulation: for example the oncogene MYC acts as a global transcriptional amplifier of cell growth and proliferation, and is implicated in many human cancers though direct MYC inhibitors remain elusive 116, 117. In a clinical study of breast cancer, the transcription factor TBX2 was shown to repress CST6 (cystatin 6), resulting in increased activity of the protease legumain (LGMN), which supports breast cancer cell proliferation and poor survival 118, 119. This is an example of a specific molecular pathway by which breast tumour cells evade homeostatic proliferation control and gain aggressive growth.

When proliferation control fails, tumour cells gain a growth advantage and subclones expand. This sets the stage for Darwinian selection: clones with highest proliferative capacity dominate, but also risk accumulating more mutations. The more rapid proliferation also may overwhelm repair mechanisms, contributing to genetic heterogeneity, a key hallmark of tumour evolution. The break of homeostasis in proliferation is a key initiating event in many tumours.

### Mechanisms of Apoptosis Evasion

Programmed cell death (apoptosis) is a critical homeostatic mechanism: it eliminates damaged, senescent or unwanted cells. In healthy tissues apoptosis balances proliferation. Tumour cells often develop resistance to apoptosis via multiple mechanisms: up-regulation of anti-apoptotic Bcl-2 family proteins, loss of pro-apoptotic proteins (Bax, Bak) 120, inactivation of death receptors, p53 pathway disruption 121, enhanced survival signalling (PI3K/Akt, NF-κB) 122, 123, and increased autophagy or senescence escape 31, 124.

In colorectal cancers with microsatellite instability, frameshift mutations in the Bax gene (pro-apoptotic) are common. Loss of Bax function contributes to resistance to anticancer treatments 125, 126. As a result, tumour cells survive despite DNA damage or therapy-induced stress, enabling the expansion of resistant clones. Also, In CLL, malignant B-cells commonly overexpress Bcl-2 (anti-apoptotic protein) and have low levels of pro-apoptotic molecules (Bax). The increased Bcl-2/Bax ratio is associated with impaired apoptosis and disease progression 125-129. Clinically, Bcl-2 inhibitors (e.g., venetoclax) have been developed to overcome this homeostasis escape.

When cells escape apoptosis, they persist even when damaged or stressed, enabling the survival of clones that would otherwise be eliminated. Surviving clones accumulate further mutations, adapt to therapy, and drive tumour progression. Apoptosis evasion is therefore intimately connected to evolutionary resilience of tumours, enabling clonal expansion under selective pressures (such as treatment or microenvironment stress).

### Changes in Cell Differentiation (Loss of Differentiation / Dedifferentiation / Anaplasia)

Differentiation is the process by which cells specialise and limit their proliferative potential; many tissues maintain homeostasis via a balance of stem/progenitor cells and differentiated cells with restricted division. Tumour cells often display dedifferentiation (loss of specialised features) or arrest at a less-differentiated (stem-like) phenotype, known as anaplasia. This deregulates homeostasis by maintaining proliferative potential, enhancing plasticity, and enabling adaptation 130-132.

Mechanistically, differentiation pathways (e.g., Notch, Wnt, Hedgehog) become dysregulated, epigenetic regulation is altered, and cancer stem-cell (CSC) phenotypes arise which contribute to heterogeneity and therapy resistance 61, 133. In APL, the t(15;17) PML-RARA fusion blocks myeloid differentiation, causing accumulation of immature promyelocytes 134. Treatment with all-trans retinoic acid (ATRA) drives differentiation and remission, illustrating how restoring homeostatic differentiation can treat cancer. In breast cancer, less differentiated (basal-like or triple-negative) tumours show worse prognosis, likely in part because the loss of differentiation confers proliferative, invasive and therapy-resistant capability. While a specific reference from the search results isn't provided here for breast differentiation, the principle is well-established in literature.

Loss of differentiation enables tumour cells to retain stem-like features, plasticity and the ability to adapt to changing environments (hypoxia, therapy, migration). This plasticity contributes to phenotypic heterogeneity, facilitates metastasis, and allows for dynamic adaptation (e.g., epithelial to mesenchymal transition, EMT). Differentiation arrest is thus another dimension by which tumour cells subvert homeostatic control.

### Interaction with the Tumour Microenvironment

Healthy tissues maintain homeostasis not only within the cells but via regulated crosstalk with the microenvironment: vasculature, stromal fibroblasts, immune cells, extracellular matrix, and metabolic milieu. Tumour cells disrupt this homeostasis by manipulating the microenvironment to favour survival, angiogenesis, immune evasion, invasion, and metastasis.

In breast cancer, anti-angiogenic therapy (e.g., VEGF/VEGFR inhibitors) can normalize tumour vasculature, improve perfusion, and enhance immune cell infiltration—thereby partially restoring homeostasis of the microenvironment and improving responses to immunotherapy 135, 136. This demonstrates clinical translation of microenvironment homeostasis manipulation. In HCC, modulation of tumour-associated macrophages is under investigation: strategies to reprogram M2-like TAMs into M1 phenotype aim to overcome immune suppression in the microenvironment and restore homeostatic immune surveillance 137-139. Some colorectal liver metastases evade anti-angiogenic therapy by *co-opting* existing hepatic vasculature rather than inducing angiogenesis 140, 141. This microenvironmental adaptation bypasses the homeostatic regulatory switch of new vessel formation and undermines therapy.

By hijacking the microenvironment, tumour cells reshape the selective pressures around them: improved nutrient supply (via angiogenesis), immune evasion (via suppression), and altered stromal context (via ECM remodelling). This allows subclones with microenvironmental advantages to dominate. Microenvironmental manipulation thus acts as a homeostatic escape route and contributes to tumour progression, metastasis and therapy resistance.

### Homeostasis Breakdown and Evolutionary Dynamics

The four major disruptions outlined: uncontrolled proliferation, apoptosis resistance, differentiation loss, and microenvironmental manipulation, represent different facets of how tumour cells evade normal cellular homeostasis. From an evolutionary viewpoint, these disruptions provide selective advantages that allow clonal expansion, competition for resources, adaptation to stress and resistance to therapy. When proliferation is deregulated, cells expand. When apoptosis is suppressed, damaged or stressed cells survive. When differentiation is lost, cells retain plasticity and adaptability. When microenvironmental manipulation occurs, cells create niches favourable to survival and growth. These combined processes allow tumour subclones to emerge, diversify, and compete, generating intratumour heterogeneity.

Treatment (chemotherapy, radiation, targeted therapy, immunotherapy) acts as a selective pressure. Tumour cells that have disrupted homeostatic controls (for example, apoptosis resistance) survive therapy and become dominant. Microenvironmental manipulation (immune suppression, hypoxia adaptation) further enables resistant clones to persist. For example, in CLL, Bcl-2 overexpressing clones resist apoptosis and are selected under therapy. In breast cancer, vascular and immune microenvironment changes select for subclones that can invade and metastasise despite therapy.

Metastatic spread involves tumour cells travelling to and colonising foreign tissue microenvironments. Successful metastasis requires adaptation of homeostasis in a new context: surviving in circulation, adhering to new stroma, inducing new vasculature, evading immune surveillance. Loss of differentiation and increased plasticity facilitate this adaptation. Tumour cells that maintain homeostatic escape mechanisms are more likely to succeed in metastasis formation.

Tumour-stroma feedback loops help re-establish tumour-specific “homeostasis” (distinct from healthy tissue homeostasis). For instance, tumour cells secrete VEGF leading to new vessels form, thus hypoxia is resolved and the tumour growth continues. The microenvironment becomes adapted to the tumour, not vice versa. Thus, tumour evolution is not simply cellular, but ecosystem-level: cells + stroma + immune + vasculature evolve together.

### Tumor Evolution and the Hallmarks of Cancer

Since the categorization of certain characteristics common to most tumors as cancer hallmarks 2,12, the major attractors in the tumor evolution process have become clear. These hallmarks represent different barriers, understood as evolutionary opportunities, that various tumors (and their microenvironment) present to promote tumor evolution toward new clones capable of bypassing these barriers and thus growing and thriving under these conditions. These hallmarks represent a new way of categorizing the disruption of homeostasis that occurs in different tumors regarding the properties that these entities predominantly acquire.

One of the most prominent examples of tumor evolution is the recurrent **activation of proliferative signaling pathways**. Within a single tumour, independent subclones frequently acquire mutations in *KRAS*, *NRAS*, *BRAF*, *EGFR*, or *PIK3CA*, even when only one such mutation is required to drive increased proliferation. Clear examples exist in colorectal cancer (CRC), where distinct metastases arising from the same patient can harbor different activating mutations in the RAS-RAF-MAPK pathway 142-144. Multiregion sequencing studies, such as those by 85 in renal cell carcinoma, demonstrated that multiple spatially separated regions independently acquired alterations in the PI3K-mTOR pathway, supporting the idea that strong selection for proliferative advantage encourages convergent evolution. The frequent recurrence of these proliferative adaptations reflects the strong evolutionary pressure to overcome cell-cycle control. Tumour microenvironments (e.g. hypoxic, nutrient-poor, and spatially constrained) favour mutations that allow cells to proliferate under adverse conditions. These conditions therefore repeatedly select for the hallmark of sustaining proliferative signaling.

Another classic hallmark that frequently arises through parallel evolution is the evasion of tumour suppressor mechanisms, especially those governed by *TP53*, *RB1*, and *CDKN2A*. Because tumour suppressors act as barriers that must be circumvented for unchecked proliferation, subclones often independently evolve diverse lesions affecting the same functional pathways. For example, 145, 146 showed that in renal cell carcinomas, multiple subclones could independently converge on inactivation of *PBRM1* or *SETD2*, both of which regulate chromatin state and genome stability. In lung adenocarcinoma, spatially segregated tumour regions frequently display different inactivating *RB1* mutations, particularly in tumours progressing toward small-cell transformation under therapeutic pressure 147. From an evolutionary standpoint, the tumour-suppressor network is redundant: disruption of one component such as *TP53* or *CDKN2A* may not fully disable growth restraint, creating ongoing selective pressure for additional, parallel disruptions. As a result, the hallmark of evading growth suppressors often emerges multiple times independently within the same tumour.

Tumour cells must evolve mechanisms to avoid apoptosis, necroptosis, and other forms of **regulated cell death**. Parallel evolution often targets key nodes such as *BCL2*, *BAX*, *CASP8*, or components of the extrinsic death receptor pathway. In head and neck squamous cell carcinoma (HNSCC), for example, different tumour regions may exhibit independent mutations or epigenetic silencing events affecting *CASP8*, which modulates death receptor signaling 148. Similarly, in melanoma, distinct metastases from the same patient can evolve independent changes in *BCL2A1* or loss of *BIM*, both of which suppress apoptosis, particularly in the context of targeted therapy 149. Therapeutic pressures powerfully shape this hallmark. Resistance to targeted therapy (e.g., BRAF inhibitors, EGFR inhibitors, hormone therapy) frequently emerges through parallel apoptotic escape routes. In EGFR-mutant lung cancer, distinct subclones independently amplify *MET* or mutate *EGFR* at T790M to bypass therapy-induced apoptosis 150, demonstrating convergent evolution in real time.

Replicative Immortality via telomere maintenance, usually by reactivation of telomerase (*TERT*) or alternative lengthening of telomeres (ALT), is another hallmark frequently achieved through parallel evolution. Mutations in the *TERT* promoter are a well-known example, occurring early in tumourigenesis but also independently emerging in spatially distinct regions of glioblastoma, melanoma, and bladder cancer 151, 152. In tumours that use the ALT pathway, distinct subclones may independently acquire loss of function in *ATRX* or *DAXX*, reflecting convergent selection for chromatin remodeling that permits ALT activation. ALT-positive osteosarcomas frequently demonstrate heterogeneous patterns of *ATRX* mutation, supporting parallel mechanisms of immortality 153. The requirement for telomere stabilization is universal: without it, tumour cells cannot proliferate indefinitely. Thus, multiple subclones may independently evolve this hallmark, particularly in highly heterogeneous or spatially segregated tumours where early clones have not yet achieved immortalization.

**Angiogenesis** offers fertile ground for parallel evolution because hypoxic gradients vary widely across tumour landscapes. As regions become hypoxic, subclones independently evolve mechanisms to induce neovascularization. Parallel upregulation of VEGF, often through mutations affecting *VHL*, *HIF1A*, or epigenetic activation of pro-angiogenic pathways, has been documented extensively, particularly in clear-cell renal cell carcinoma (RCC), where VHL loss is nearly universal. Yet even within VHL-deficient tumours, independent subclones may further enhance HIF signaling through convergent mutations in pathway regulators 154. This hallmark is tightly linked to the tumour microenvironment. The fluctuating oxygenation levels across different tumour niches create repeated, parallel selection pressures. A region that becomes hypoxic later in progression may select for angiogenic drivers even if other regions already evolved such capabilities.

The **transition to invasive or metastatic phenotypes** can also arise through parallel evolution. While once thought to be a late, linear step, multiregion sequencing has demonstrated that metastases can seed early and evolve in parallel to the primary tumour 155. Independent metastatic lineages may each acquire distinct mutations in genes promoting epithelial-mesenchymal transition (EMT), motility, or extracellular matrix remodeling, such as *CDH1*, *PTEN*, *NF2*, or regulators of the Rho GTPase network. For example, in triple-negative breast cancer, different metastatic lesions can develop independent inactivating mutations in *PTEN* that enhance invasive behavior and metastatic potential 156, 157. Similarly, in pancreatic cancer, independent metastatic subclones may converge on inactivation of *SMAD4*, facilitating dissemination and colonization of distant organs 93. Spatially constrained subpopulations in primary tumours may face differential pressures to invade based on stromal composition, vascular proximity, or immune infiltration. These pressures create multiple evolutionary paths toward the hallmark of invasion and metastasis.

**Metabolic reprogramming** is another arena where convergent evolution occurs. Tumours frequently shift toward glycolysis (the Warburg effect), glutamine addiction, or fatty-acid metabolism. Genomic changes supporting this shift, such as *IDH1/2* mutations, alterations in *MYC*, or upregulation of glycolytic enzymes, may evolve independently in different tumour regions. In gliomas, distinct subclones sometimes independently acquire *IDH1* mutations, although this is less common due to the early timing of IDH mutations in many cases. More commonly, parallel evolution affects metabolic regulators such as *PTEN*, *PIK3CA*, and *AMPK* pathway components, which collectively reshape nutrient uptake and utilization 87.

**Genome instability** both drives and is reshaped by parallel evolution. Tumours often acquire independent inactivating mutations in DNA damage response (DDR) genes, including *BRCA1/2*, *ATM*, *CHEK2*, or mismatch repair genes. This phenomenon is well-described in high-grade serous ovarian cancer, where parallel inactivation of homologous recombination genes can arise across different tumour deposits 158. As genomic instability increases, the evolutionary search space broadens. This often accelerates the rate at which other hallmarks evolve in parallel, creating feedback between instability and phenotypic convergence.

Immune-Mediated tumor evolution and immune escape usually is presented in 4 different forms: *1. Loss of Antigen Presentation.* Tumor cells frequently evade immune surveillance through genetic and epigenetic alterations that impair antigen presentation. Loss-of-function mutations or deletions in HLA class I genes, β2-microglobulin (B2M), and components of the antigen-processing machinery reduce neoantigen visibility and confer resistance to cytotoxic T cell-mediated killing 21, 51, 159. Such alterations often arise under immune pressure and are enriched in tumors exposed to immune checkpoint blockade, highlighting immune escape as a selectable evolutionary trait. *2. Disruption of Interferon Signaling Pathways.* Interferon (IFN) signaling plays a central role in anti-tumor immunity by promoting antigen presentation and immune cell recruitment. Tumors can acquire mutations in IFN pathway components, including JAK1, JAK2, and STAT1, thereby rendering cancer cells insensitive to IFN-mediated growth arrest and immune activation. These alterations have been repeatedly identified in tumors that relapse after immunotherapy, illustrating therapy-driven immune selection. For example, 160 and 161 reported acquired JAK1/JAK2 mutations in melanoma progressing after anti-PD-1 therapy, implicating IFN pathway disruption in clinical immunotherapy resistance. Therefore, IFN-JAK/STAT pathway alterations serve as biomarkers for primary or acquired ICI resistance and can inform therapeutic stratification and combination strategies. *3. Immune-Excluded and Immunosuppressive Microenvironments.* Tumors often show immune cells (e.g., CD8⁺ T cells) at the peritumoral margin but limited infiltration into tumor nests, which correlates with poor clinical response to ICIs and is a hallmark of immune escape. Spatial transcriptomics and single-cell profiling technologies reveal that the tumor microenvironment (TME) can be highly heterogeneous within the same tumor mass, with regional differences in immune infiltration and activation states. Such local microenvironmental pressures can drive immune-tumor co-evolution. For example, distinct stromal and vascular features (e.g., dense extracellular matrix, abnormal vasculature, and hypoxic regions) are associated with T-cell exclusion and immunosuppressive signals. In some NSCLC patients with ICI resistance, upregulation of alternate immunosuppressive checkpoints and exclusionary phenotypes (T-cells restricted to invasive margins) have been detected in relapsed tumors, supporting immune exclusion as part of acquired immune escape 162, 163, 164. Immune exclusion and suppressive TMEs explain failure of T-cell infiltration, support the need for combination therapies targeting stroma, vasculature, or hypoxia, and underscore regional selective pressures within tumors as clinically meaningful.

*4. Therapy-Driven Immune Selection and Neoantigen Evolution.* Matched pre-treatment and resistant tumors from NSCLC patients treated with anti-PD-1 (alone or with anti-CTLA-4) demonstrated loss of multiple neoantigens in resistant clones. Loss occurred via deletion of chromosomal regions or elimination of tumor subclones harboring immunogenic neoantigens, consistent with immune selection and immunoediting 162, 163, 165, 166. Neoantigen loss in resistant tumors was associated with changes in T-cell receptor clonality and reduced immunogenicity, underscoring that neoantigen landscape evolution is a dynamic process during ICI therapy, rather than a static predictor.

The immune system (endogenous or therapy-augmented) exerts selection against highly immunogenic neoantigens, leading to clonal extinction or outgrowth of less immunogenic subclones that evade detection. Neoantigen dynamics contribute to adaptive resistance; strategies like longitudinal ctDNA/neoantigen monitoring and neoantigen-targeted therapies may improve evolutionary forecasting and personalized immunotherapy.

Immune-mediated selection operates across distinct temporal phases of tumor evolution. Early immune pressure can shape tumor initiation by eliminating highly immunogenic clones, a process termed immunoediting. In contrast, late-stage tumors, particularly under therapeutic intervention, experience intensified and focused immune selection that promotes genetic immune escape and phenotypic adaptation. Recognizing these temporal layers is critical for understanding why immunotherapy responses differ between early and advanced disease.

## Clinical Implications

In summary, the Darwinian evolution of a tumor involves the accumulation of mutations and epigenetic changes in cancer cells over time, allowing them to acquire characteristics that allow them to evade normal controls of cellular homeostasis and thrive in a hostile environment. This process of tumor evolution reflects a constant struggle between cancer cells and the body's regulatory mechanisms, which often results in the adaptation of tumor cells to promote their survival and growth at the expense of normal tissue and function of the body. Tumor evolution leads to treatment resistance due to the high genetic and adaptive plasticity of cancer cells and therefore, drug adaptation may be considered a transition of progressively adaptative cellular states that eventually turn into stable resistance 167. There are several reasons why tumors tend to develop resistance to treatments:

As mentioned before, tumors are highly heterogeneous, meaning they contain subpopulations of cells with different genetic and phenotypic profiles (Table 1). This genetic diversity provides a reservoir of cells that can survive and proliferate under the pressure of treatments, allowing the tumor to evolve and develop primary resistance. However, apart from those pre-existing clones, cancer cells can acquire additional genetic mutations, while being exposed to different drugs, which give them resistance to treatments. For example, they can develop mutations in the genes that encode therapeutic targets, such as the epidermal growth factor receptor (EGFR) in advanced non-small cell lung cancer (NSCLC), making it difficult for drugs (EGFR tyrosine kinase inhibitors, in this case) to bind to their target and exert their effect. Moreover, different drugs are likely to lead to different mutations and acquired resistance can happen through on- or off-target mechanisms, reducing the drug's ability to inhibit the signaling pathway 168, 169.

Additionally, cancer cells can activate alternative signaling pathways that allow them to survive and proliferate even in the presence of treatments that are designed to inhibit specific pathways. Those signaling pathways are intracellular communication systems that regulate a wide variety of cellular processes, such as cell proliferation, survival, differentiation, and death. These pathways are typically regulated by the interaction of proteins, such as membrane receptors, intracellular signaling proteins, and transcription factors, which transmit signals from the outside of the cell to the nucleus. In the context of cancer, malignant cells often depend on specific signaling pathways to maintain their uncontrolled proliferation and survivability so that their ability to "escape" the effect of specific treatments contributes to acquire resistance. A case in point is the cell's intrinsic adaptive response to treatments which target the well-known PI3K/AKT/mTOR pathway or the epidermal growth factor (EGFR) pathway. When these signaling pathways are inhibited by kinase inhibitors or EGFR inhibitors respectively, cell compensatory mechanisms and parallel pathways (e.g MAPK/ERK pathway or IGF-R1 pathway) get activated in order to reactivate the main pathway through feedback loops and to cancer cells to continue proliferating, eventually giving rise to therapeutic efficacy reduction 170 171.

Another key compensatory bypass mechanism of resistance to targeted therapy is the increased expression or activity of specific receptors on the cell surface, allowing cancer cells to continue receiving growth and survival signals even in the presence of treatment. For example, Khelwatty SA *et al*. 172, reported that DiFi colorectal cancer cells can increase the expression of cell surface EGFR and the phosphorylation of HER-2 and HER-3 (upregulation) to compensate for the inhibition of this signaling pathway, while treating them with chronic doses of anti-RGFR antibody ICR62 or the EGFR tirosyn kinase inhibitor, gefitinib 172.

Furthermore, not only can the tumor microenvironment provide protection and support to cancer cells, facilitating their survival and metastasis, but also plays a relevant role in the development of resistance to treatments. For instance, the formation of new blood vessels (angiogenesis) can impact the delivery of oxygen, nutrients and drugs to the tumor, making it more resistant to therapy and often indicating a poor clinical prognosis in some patients. So much so that drugs targeting angiogenesis have shown clinical benefits when combined with other therapies, such as chemotherapy, radiotherapy or immunotherapy 173.

In addition to genetic mutations, cancer cells can undergo epigenetic changes that alter gene expression and cellular function. These epigenetic changes may contribute to treatment resistance and tumor relapse by regulating the cellular response to drugs and the surrounding environment. For example, epigenetic dysregulation commonly occurs in endocrine-resistant breast cancer and epigenetic adaptation is associated with therapy-induced dormancy in ER+ breast cancer, causing persistent cancer cell population and reducing drug's cytotoxic effects 25 174.

Taking all of the former into account, cancer cells can activate survival pathways that allow them to resist the effects of treatment and avoid cell death. This plasticity and adaptability of cancer cells are important challenges in cancer treatment and underscore the importance of developing more effective therapies that address cancer heterogeneity and plasticity.

Recent clinical studies demonstrate that evolutionary metrics, such as clonal diversity, mutational burden dynamics, and subclonal architecture, can predict prognosis, therapy response, and resistance across cancer types 175, 176. These findings support the clinical relevance of evolutionary-informed treatment strategies. Recent conceptual analyses further emphasize the importance of clearly defining evolutionary units, traits, and selection pressures in cancer, reinforcing the need for integrative and multi-scale models 177.

## Evolution-Informed Treatment Strategies

### 1. Combination Therapy: Limiting Evolutionary Escape

Combination therapies aim to reduce the probability of resistance by simultaneously targeting multiple vulnerabilities, thereby constraining the evolutionary space available to tumor cells. From an evolutionary perspective, this strategy exploits the low likelihood that a single clone can harbor resistance mechanisms to multiple agents simultaneously.

Required biomarkers/monitoring: Baseline clonal composition, pathway activation states, and resistance-associated alterations assessed by bulk or single-cell profiling.

Limitations: Increased toxicity, selection for broadly resistant or plastic phenotypes, and potential elimination of sensitive competitors that suppress resistant clones.

### 2. Sequential Therapy: Steering Evolutionary Trajectories

Sequential treatment strategies leverage the concept that resistance to one therapy may induce collateral sensitivity to another. By ordering therapies appropriately, tumor evolution can be steered toward less aggressive or more treatable states.Required biomarkers/monitoring: Longitudinal molecular profiling (e.g., ctDNA), pathway rewiring signatures, and resistance mechanism identification. Limitations: Requires accurate prediction of evolutionary trajectories; mis-sequencing may accelerate resistance.

### 3. Adaptive (Evolutionary) Therapy: Maintaining Competitive Suppression

Adaptive therapy seeks to control, rather than eradicate, tumors by maintaining a population of therapy-sensitive cells that suppress the expansion of resistant clones. This approach is grounded in ecological competition and frequency-dependent selection. Required biomarkers/monitoring: Real-time tumor burden assessment, ctDNA-based resistance tracking, and dynamic treatment adjustment. Limitations: Risk of under-treatment, need for frequent monitoring, and uncertain applicability across tumor types.

### 4. Ecosystem-Targeting Approaches: Modifying the Selective Environment

Rather than directly targeting tumor cells, ecosystem-based strategies aim to alter the selective pressures imposed by the tumor microenvironment, including hypoxia, immune exclusion, stromal interactions, and nutrient availability. By reshaping the ecosystem, these approaches seek to reduce the adaptive advantage of aggressive or stem-like phenotypes. Required biomarkers/monitoring: Spatial profiling, hypoxia markers, immune infiltration metrics, and microenvironmental signatures. Limitations: Tumor ecosystems are heterogeneous and dynamic; compensatory pathways may emerge.

### Failure and Practical Constraints of Evolutionary Therapies

Despite their promise, evolution-informed therapies face important limitations. Tumors may evolve along unanticipated trajectories, exploit non-targeted resistance mechanisms, or leverage phenotypic plasticity to bypass selective constraints. In addition, logistical challenges—including the need for frequent monitoring, complex decision algorithms, and patient compliance—may limit clinical implementation. These considerations underscore the need for prospective trials explicitly designed to test evolutionary principles.

**Figure 5:**
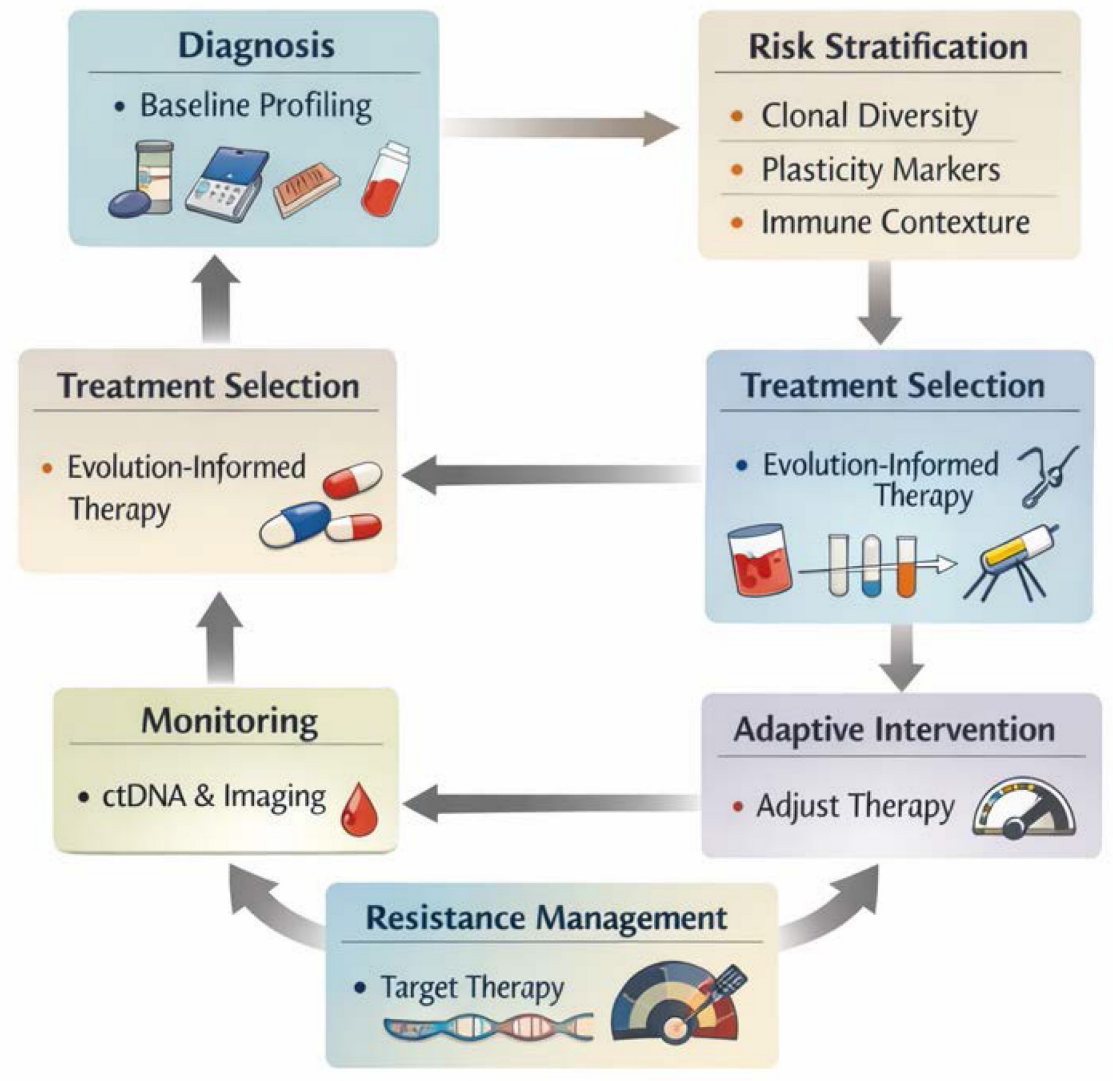
Evolution-informed clinical management of cancer*.* Cancer treatment becomes a dynamic, adaptive process guided by evolutionary monitoring rather than a fixed intervention.

Ultimately, effective cancer control will likely require integrating evolutionary principles into treatment design, monitoring, and adaptation, transforming therapy from a static intervention into a dynamic process informed by tumor evolution (Figure 5). The integrative framework and its clinical implications are summarized in Figures 4 and 5 and Table 2, which provide a visual synthesis of tumor evolution as a dynamic and actionable process.

## Conclusions

Tumour evolution can be viewed most coherently as a gradual and multifaceted disruption of tissue homeostasis, in which cancer cells progressively evade the regulatory systems that normally govern proliferation, differentiation, cell death and microenvironmental balance. By loosening these constraints, malignant cells gain the flexibility to initiate tumour growth, expand across diverse ecological niches, and persist under therapeutic stress. This process is not linear; it is shaped by substantial intratumour heterogeneity, with different subclones exploiting distinct escape strategies, and by cellular plasticity, which allows tumours to shift phenotypes, remodel their surroundings and survive otherwise lethal conditions. Even when therapies effectively target specific vulnerabilities (such as aberrant signalling pathways, apoptotic defects or stromal support) resistance commonly emerges. It may be present from the outset (primary resistance), or arise through evolutionary selection during treatment (acquired resistance), driven by genetic diversification, pathway rewiring, or adaptive changes in the tumour microenvironment.

Looking forward, more effective cancer control will depend on anticipating and intercepting these dynamic processes rather than reacting to them after resistance has emerged. This requires a deeper understanding of how tumour cells coordinate multiple homeostatic escape mechanisms simultaneously, and how these mechanisms shift under therapeutic pressure, during metastatic spread, or within protective microenvironmental niches. New technologies such as single-cell and spatial multiomics, multi-region tumour sampling, dynamic liquid biopsies and computational evolutionary modelling are beginning to reveal these interactions with unprecedented resolution. Integrating this knowledge into clinical practice will rely on robust biomarker development, personalised therapeutic design, and combination strategies that simultaneously target cell cycle control, apoptosis, immune modulation and stromal adaptation. Ultimately, viewing cancer through the lens of homeostasis not only unifies disparate biological and clinical observations, but also suggests that restoring, stabilizing or re-engineering tissue balance, rather than focusing on isolated tumour traits, may provide a more durable and conceptually coherent path toward preventing progression, delaying resistance and improving long-term patient outcomes.

## Figures and Tables

**Figure 1:**
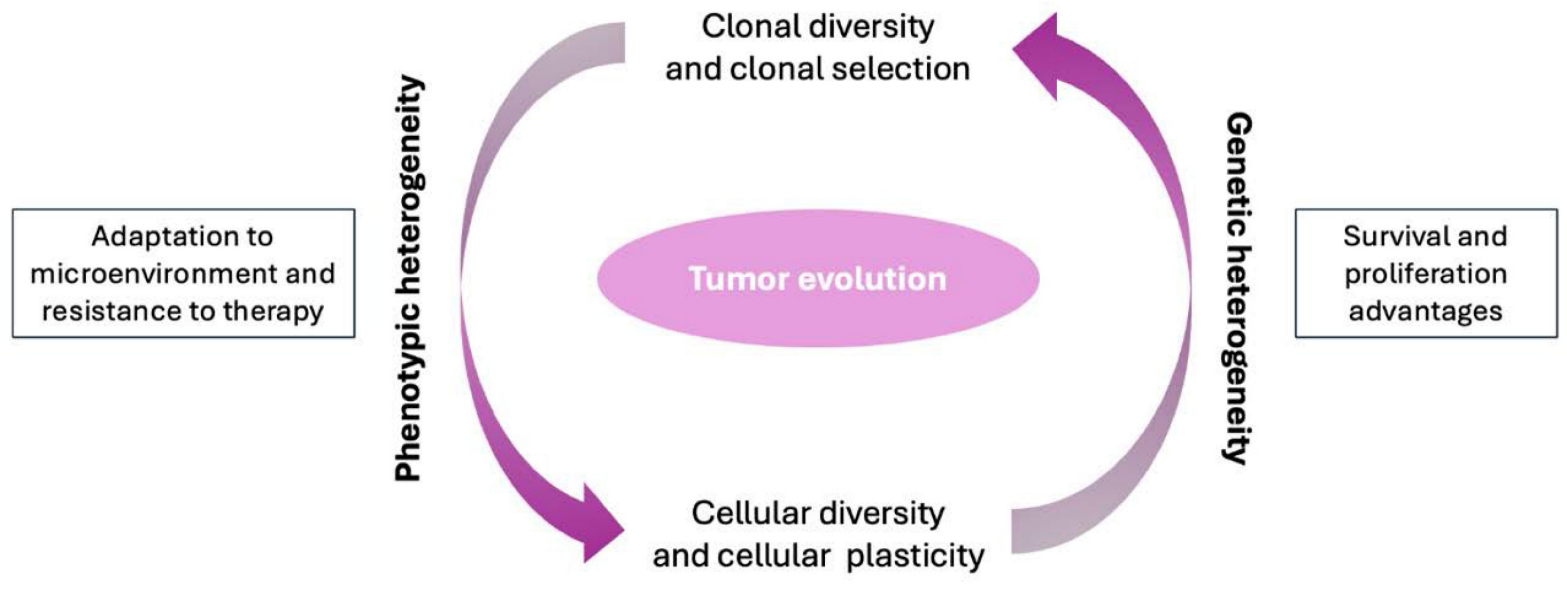
Conceptual framework of tumor evolution as a dynamic entity integrating genetic and phenotypic heterogeneity, spatial organization, and microenvironmental selection pressures, extending classical Darwinian models.

**Figure 2:**
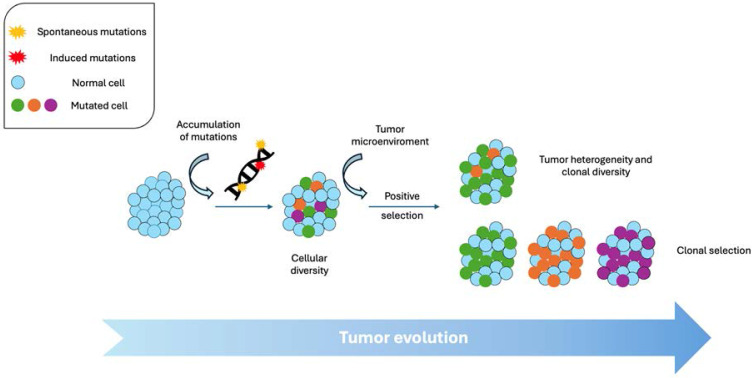
General scheme of clonal diversity during tumor evolution.

**Figure 3:**
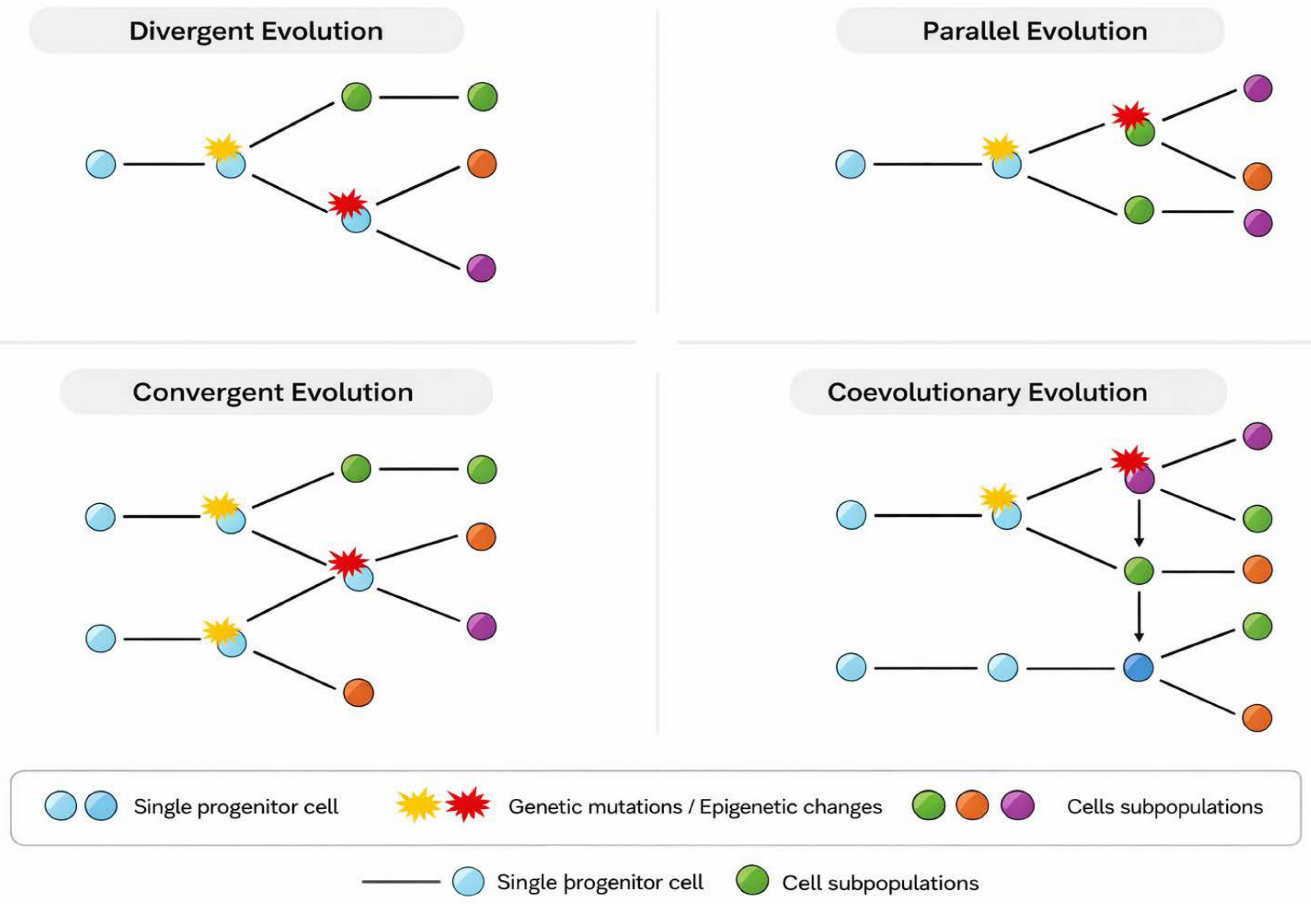
General types of tumor evolution.

**Table 1:** ** Experimental and clinical approaches to infer tumor evolutionary dynamics.** Summary of major experimental and clinical approaches used to infer tumor evolutionary dynamics, highlighting the types of evolutionary processes that can be reconstructed and the principal limitations of each methodology.

Data modality	Evolutionary inferences enabled	Key strengths	Major limitations
Bulk tumor sequencing	Clonal architecture, driver mutations, mutational burden	Widely available; clinically integrated	Averages signals across cells; limited resolution of subclones
Multi-region tumor sampling	Spatial heterogeneity, branched vs linear evolution, early vs late events	Reveals regional diversification	Invasive; limited sampling density
Single-cell genomics / transcriptomics	Clonal diversity, lineage relationships, cell-state plasticity	High-resolution genetic and phenotypic mapping	Costly; limited clinical scalability
Spatial transcriptomics / proteomics	Local adaptation, microenvironment-tumor co-evolution	Preserves tissue context	Lower depth than single-cell; complex analysis
Circulating tumor DNA (ctDNA)	Temporal evolution, treatment response, emerging resistance	Minimally invasive; longitudinal monitoring	Limited sensitivity for low-burden disease

**Table 2:** Evolutionary modes in cancer: phenotypic outcomes, measurement strategies, and therapeutic implications

Evolutionary mode	Typical phenotypic outcome	Measurement strategies	Therapeutic implications
Linear evolution	Stepwise progression	Bulk sequencing	Target dominant clone
Branched evolution	High intratumour heterogeneity	Multi-region sequencing	Combination therapies
Neutral evolution	Genetic diversity without selection	Variant allele frequency analysis	Limited predictive biomarkers
Punctuated (“Big Bang”) evolution	Early diversification	Early multi-region sampling	Importance of early intervention
Convergent/parallel evolution	Recurrent pathway activation	Phylogenetics, pathway analysis	Target common dependencies
Phenotypic plasticity	Drug tolerance, stemness	Single-cell & spatial omics	Target regulators of plasticity
Immune-driven evolution	Immune escape variants	Neoantigen tracking, spatial profiling	Immunotherapy combinations
